# The Physical Activity and Sedentary Behaviour Patterns of Children in Kindergarten and Grade 2

**DOI:** 10.3390/children5100131

**Published:** 2018-09-20

**Authors:** Jeff R. Crane, Patti-Jean Naylor, Viviene A. Temple

**Affiliations:** School of Exercise Science, Physical and Health Education, University of Victoria, Victoria, BC V8P 5C2, Canada; jrcrane@mun.ca (J.R.C.); pjnaylor@uvic.ca (P.-J.N.)

**Keywords:** physical activity, sedentary, health, accelerometer, children, transitions

## Abstract

Accompanying the transition from early to middle childhood are substantial changes in children’s educational and recreational circumstances. These changes may affect physical activity levels. This study examined levels of physical activity and sedentary behaviours in kindergarten (age range 5–6 years) and grade 2 (age range 7–8 years). Participants were 96 kindergarten children recruited in the 2010–2011 and 2011–2012 school years and 94 grade 2 children recruited in the 2012–2013 and 2013–2014 school years. A sub-cohort of children was tracked longitudinally from kindergarten to grade 2. Accelerometers were used to measure physical activity and sedentary behaviour. Independent *t*-tests revealed that children in grade 2 spent significantly less time engaged in physical activity compared to those in kindergarten (292 min/day compared with 354 min/day) and more in sedentary behaviours (443 min/day compared with 368 min/day). For the longitudinal sample, the pattern was similar. Repeated measures ANOVA revealed a significant decrease in physical activity levels (364 min/day to 292 min/day) and a significant increase in sedentary behaviour (368 min/day to 435 min/day) over time. There is a critical need to invest in strategies to maintain higher levels of physical activity across the primary years and reduce sedentary time, since these behaviours affect health outcomes.

## 1. Introduction

The Canadian 24-h movement guidelines for children and youth recommend that children 5–17 years of age accumulate a minimum of 60 min of moderate-to-vigorous physical activity (MVPA) per day to achieve health benefits [1]. Additionally, children should engage in vigorous activities as well as activities to strengthen muscles and bones three or more days per week. Nationally representative data reveal that approximately 7% of Canadian 5–11–year-old children meet daily MVPA guidelines [2,3]. However, when fewer days per week were examined, 40% and 80% of Canadian children achieved the recommended daily 60 min of MVPA at least three days and at least one day per week, respectively [4]. While these data illustrate that 60 min of MVPA in a single day is achievable for Canadian children, consistency continues to be problematic.

Consistent engagement in MVPA has gained considerable attention in the physical activity and public health literature because of the health benefits associated with regular participation [4,5,6,7,8,9]. Regular physical activity has musculoskeletal and cardiovascular health benefits and assists with the maintenance of healthy body weight among children [4,8,10]. Furthermore, regular physical activity decreases the likelihood of becoming overweight or obese by as much as 70% [11] and engaging in MVPA (e.g., brisk walking) has been linked to reductions in blood pressure among children and youth [10]. In contrast, engagement in sedentary activities (e.g., television watching) significantly increases the likelihood of becoming overweight or obese by as much as 61% [11] and has been positively correlated with being at risk of metabolic syndrome and hypertension [12,13,14].

To date, evidence of age-related trends in physical activity participation during childhood has consisted of both cross-sectional and longitudinal studies. Cross-sectional studies have shown that the physical activity levels of younger children (3–5 years of age) are higher than children in middle/late childhood and adolescence, and levels of sedentary behaviour are lower [15,16,17,18,19]. Similarly, longitudinal studies [20,21,22,23], as well as a systematic review [24] examining physical activity and sedentary behaviour over time, highlight a decline in physical activity and increase in sedentary behaviour with age.

In Canada, cross-sectional findings show 14% of 5-year-olds [3] and 9% and 4% of 6–10-year-old boys and girls [2] accumulated the minimum 60 min of MVPA. These two studies also revealed that 5-year-old children spent less time engaged in sedentary behaviour when compared to children 6–10 years of age. The trend toward lower levels of physical activity with increasing age is also apparent from middle childhood, to adolescence, to adulthood. For example, cross-sectional findings from Troiano and colleagues [25] found that 42% of children aged 6–11 years accumulated the recommended amount of at least one hour of physical activity per day, whereas only 8% of adolescents and 5% of young adults met the guideline [25].

Jones and colleagues’ [24] systematic review highlights the variability in tracking coefficients found across early and middle childhood. They report that 83% of studies found moderate or better levels of sedentary behaviour tracking compared to the 64% that reported moderate or better levels of tracking for total physical activity [24]. The inconsistencies in the level of tracking reported for physical activity and sedentary behaviour may be related to the methods and tools used to collect these data. For example, modest levels of tracking were reported using accelerometers in early childhood [22] compared to Pate et al. [26] who found physical activity to track reasonably well using the PAHR-50 index (activity > 50% of resting heart rate). For middle childhood, Janz et al. [21] found moderate levels of tracking for physical activity and sedentary behaviour in comparison to modest levels of tracking for physical activity with pedometers. The discrepancies among tools used to collect both physical activity and sedentary data may have an influence on the levels of tracking when comparing results between studies.

Few studies have concurrently examined both physical activity and sedentary behaviours of children longitudinally. Perhaps this is because of some of the difficulties associated with longitudinal research, mainly drop out or failure to meet inclusion criteria. Jones and colleagues [24] identified only two studies (out of fourteen) that reported both physical activity and sedentary behaviour tracking coefficients [21,22]. Janz and colleagues [21] had the lone study that tracked both physical and sedentary behaviour among school-aged children over three years (from 5 to 8 years of age). At follow-up, Spearman rank-order correlation coefficients revealed that the children’s sedentary behaviour tracked more consistently (*r* = 0.37–0.52) than total activity (*r* = 0.18–0.39). It has been more than a decade since Janz and colleagues collected their data using 60-s epoch lengths and three metabolic equivalent (3 MET) cut-points for MVPA. More recently, the consensus is that when measuring physical activity among children using accelerometry, researchers should use shorter epoch lengths and a 4 MET cut-point for MVPA [27,28].

How MVPA is classified is a major methodological issue. Using a threshold count that is too low may overestimate the amount of time spent in MVPA [28,29]. The cut-off for MVPA typically used with adults is 3 METs [30], however, several studies with children have shown that brisk walking, which is a form of moderate-intensity activity, requires an energy expenditure of 4 METs [28,31]. A secondary methodological issue is epoch length. An epoch is a filtered digitized acceleration signal over a user-specified time interval, which sums counts of movement, which is typically in counts per minute (1-min epochs) [28,31]. However, researchers have concluded that unlike adults, children’s physical activity patterns are usually sporadic and intermittent in duration, typically lasting several seconds; as such, researchers have suggested that using 1-min epochs may result in an underestimation of participation in MVPA [28,32]. Therefore, measuring MVPA at 4 METs and using 15-s epoch lengths are more likely to yield more accurate results.

The present study extends the work of Janz and colleagues [21] by using cut-points (specifically, 4 METs for MVPA) and sampling rates (15-s epochs) that should produce a more accurate estimate of children’s physical activity. Additionally, reporting MVPA calculated at 3 METs allows for comparison with previously published studies where 3 METs were used as a cut-point, and measuring both physical activity and sedentary behaviour provides a more complete picture of how children spend the majority of their day. Therefore, the aim of this study was to examine the levels of physical activity and sedentary behaviours sequentially from kindergarten to grade 2 cross-sectionally and in a small sub-cohort longitudinally. Specifically, we hypothesized that: (1) children’s MVPA and total physical activity levels would be higher in kindergarten compared to grade 2, and (2) sedentary behaviour would be lower in kindergarten compared to grade 2. To contribute methodologically to the literature, we also examined whether the differences between MVPA measured at 3 METs and 4 METs would be meaningful and whether the findings from the cross-sectional sample would be different to those collected in the longitudinal sample.

## 2. Materials and Methods

The University of Victoria Human Research Ethics Board and the school district granted approval for this study. Data were collected in sequence over a period of four school years from 2010 to 2014 and included a cross-sectional and a longitudinal sample. The University ethics protocol number is 10-246, and the original ethics approval date was 23 June 2010. As per Canadian federal regulations, our ethics protocol was renewed in each school year.

### 2.1. Participants

Children were eligible to participate if they were attending one of eight consenting schools from one school district in the province of British Columbia, Canada, and if their parent(s)/guardian(s) provided informed consent to the study. In this school district, results of provincial screening using the Early Development Index, a measure estimating school readiness in kindergarten, demonstrated that rates of Physical Health and Well Being, Social Competence, Emotional Maturity, Language and Cognitive Development, and Communication Skills, were higher than or equivalent to provincial rates [33].

The first kindergarten cohort (wave one) was collected during the 2010–2011 school year and the second cohort of kindergarten children (wave two) during the 2011–2012 school year. In grade 2, data on cohort one participants were collected during the 2012–2013 school year and during the 2013–2014 school year for cohort two. Of the 206 children that consented to participate in kindergarten, 96 (Mean age = 5 years 7 months, 58% boys, Mean Body Mass Index (BMI) = 15.5 ± 1.8) met the accelerometer wear time criteria of 10 h of recorded physical activity and sedentary behaviour on at least three weekdays and one weekend day. In grade 2, 94 participants (Mean age = 7 years 9 months, 52% boys, Mean BMI = 15.8 ± 2.1) met the accelerometer inclusion criteria of the 185 recruited. Twenty-one of those children (49.6% boys) had valid physical activity and sedentary behaviour data for both kindergarten and grade 2 and comprised the longitudinal sample. The comparative cross-sectional sample was therefore 75 (96 − 21) in kindergarten and 73 (94 − 21) in grade 2. The total sample was *n* = 190 individuals.

### 2.2. Measures

Physical activity levels were measured using the Actigraph GT1M accelerometer (ActiGraph, LLC, Fort Walton Beach, FL, USA). This was worn around the waist and positioned above the iliac crest on the right hip and recorded both frequency and intensity of acceleration or movement. This uniaxial accelerometer collected pre-filtered data at a rate of 30 measurements per second (30 Hz), which was then post-filtered into epochs. In this study, 15-s epochs were used to record physical activity and then converted to a metabolic equivalent (MET). Fifteen-second epochs have been recommended in order to record the sporadic activity of younger children [34]. The Actigraph accelerometer has been shown to be a valid indicator of energy expenditure and activity levels in children and youth [35,36,37]. Average wear time for children included in the study in kindergarten was 12.0 h (*Std Deviation* = 0.8) and in grade 2 was 12.1 h (*Std Deviation* = 0.7). Sixteen percent of included children met the weekday wear-time criteria on three weekdays, 67% on four weekdays, and 17% on five weekdays; 50% of children met the weekend wear-time criteria on one weekend day and 50% on two weekend days.

### 2.3. Procedures

Each child was fitted with an accelerometer during school hours and an information package was sent home to the parents/guardians. Parents were asked to have their child wear the accelerometer from when the child rose in the morning until they went to bed, only to be removed when swimming or bathing. This procedure was the same in kindergarten and grade 2. Data were collected in the same season in kindergarten and grade 2 (ranging from fall to spring) for each school. For example, if accelerometry data were collected in the spring in kindergarten at a particular school, data were collected in spring in grade 2 at that school.

The minimum valid wear time was 10 h per day for at least four days (including at least three weekdays and one weekend day) [38]. Based on comparable studies [28,39], the recorded physical activity and sedentary behaviour were classified by intensity into the following categories: MVPA as ≥4 METs and as ≥3 METs; total physical activity as ≥1.5 METs; and sedentary behaviour as <1.5 METs. MVPA was classified at both ≥3 METs and ≥4 METs because although ≥4 METs is a more accurate cut-point for MVPA in children [31,40], many studies have classified children’s MVPA at ≥3 METs [2,3,21,41]. Including ≥3 METs allowed for easier comparisons with the existing literature. Both light and MVPA were summed to represent total physical activity. In order to standardize the minutes of physical activity and sedentary behaviours accrued, activity minutes were adjusted to minutes per hour and all analyses used this measure.

### 2.4. Data Treatment and Analyses

Raw data from the accelerometers were downloaded using ActiLife software (Actigraph LLC) for subsequent data reduction. Kinesoft software (version 2.0.94, Kinesoft Software, Rothesay, NB, Canada) was used for analysis, extraction, and processing of the physical activity data. Kinesoft age-specific cut-points were used, specifically: in kindergarten, sedentary behaviour ≤ 150 counts per minute, 3 METs ≥ 614 counts per minute, and 4 METs ≥ 1400 counts per minute. In grade 2, the age-specific cut-points were: sedentary behaviour ≤ 185 counts per minute, 3 METs ≥ 705 counts per minute, and 4 METs ≥ 1515 counts per minute. Data analyses were performed using IBM SPSS (Version 23.0) for Windows [42]. Descriptive statistics were computed for average minutes of physical activity per day and minutes per hour at the following intensities: sedentary, MVPA, and total. Multiple independent *t*-tests were computed to examine any differences in physical activity and sedentary behaviour among the kindergarten and grade 2 samples as well as to determine whether there were any differences between the cross-sectional and longitudinal samples. Repeated measures analyses of variance (ANOVA) using grade level as the between-subject factor were used to examine the differences in physical activity and sedentary behaviour in the longitudinal sample.

## 3. Results

### 3.1. Cross-Sectional Physical Activity and Sedentary Behaviour

Table 1 and Table 2 display the cross-sectional descriptive statistics for physical activity (MVPA and total physical activity) and sedentary behaviour in minutes per day and minutes per hour, respectively. Physical activity levels were generally high for children in both kindergarten and grade 2. Furthermore, in each grade, children accumulated more minutes in sedentary behaviours than either MVPA or total physical activity. For the entire cross-sectional sample of 148 participants, independent *t*-tests revealed that physical activity levels were significantly lower among the grade 2 students and sedentary behaviour was significantly higher. Specifically, MVPA *t* (141) = 6.881, *p* < 0.001) and total physical activity *t* (141) = 8.364, *p* < 0.001) was lower by grade 2, whereas sedentary behaviour was significantly higher in grade 2 *t* (141) = −9.313, *p* < 0.001). An independent samples *t*-test revealed there were no significant differences between the boys and girls in either kindergarten or grade 2.

### 3.2. Longitudinal Physical Activity and Sedentary Behaviour

Table 3 and Table 4 provide the descriptive statistics for physical activity (MVPA and total physical activity) and sedentary behaviour in minutes per day and per hour for the longitudinal sample. Overall, physical activity levels were high in both kindergarten and grade 2 but declined over time. Sedentary behaviour accounted for more minutes than total physical activity and increased by grade 2.

Repeated measures ANOVA revealed significant differences in both physical activity levels and sedentary behaviour over time in the longitudinal sample (*n* = 21). Specifically, Wilks’ lambda (λ) = 0.21, *F* (1,20) = 74.21, *p <* 0.001 for sedentary behaviour, λ = 0.36, *F* (1,20) = 34.88, *p* < 0.001 for MVPA, and λ = 0.35, *F* (1,20) = 35.72, *p* < 0.001 for total activity show that sedentary behaviour increased significantly while both MVPA and total physical activity decreased from kindergarten to grade 2. Pearson product-moment correlations revealed that sedentary behaviour in kindergarten was significantly correlated (*r* = 0.56, *p* < 0.01) with sedentary behaviour in grade 2. Both MVPA (*r* = 0.23, *p* < 0.05) and total physical activity (*r* = 0.32, *p* < 0.05) tracked from kindergarten to grade 2. The intra-class correlation coefficients comparing kindergarten and grade 2 min were: 0.59 for total physical, 0.30 for MVPA, and 0.77 for sedentary behaviour.

### 3.3. Differences between the Cross-Sectional Sample and Longitudinal Sample

Independent *t*-tests revealed that there were no significant differences between the cross-sectional sample and longitudinal sample in either kindergarten or grade 2. In kindergarten, we found no significant differences for MVPA *t* (94) = 0.743, *p* = 0.254, total physical activity *t* (94) = 0.950, *p* = 0.315, and sedentary behaviour *t* (94) = 0.818, *p* = −0.831. Similarly, we found no significant differences between the grade 2 samples for MVPA *t* (94) = 0.011, *p* = 0.729, total physical activity *t* (92) = 0.061, *p* = 0.938, or sedentary behaviour *t* (92) = −0.811, *p* = 0.347.

## 4. Discussion

We set out to examine differences in physical activity levels and sedentary behaviour from kindergarten to grade 2. Our findings highlight the decline in physical activity minutes and increase in sedentary behaviours cross-sectionally and with a longitudinal sub-cohort. Our findings contribute to a growing body of evidence surrounding the issues of PA and sedentary behaviour, and our study is one of only a few studies to explore these factors over time, which is discussed further throughout the discussion.

### 4.1. Physical Activity and Sedentary Behaviour

Overall, the physical activity levels of children in kindergarten and grade 2 were high. Kindergarten and grade 2 children accumulated an average of 133 and 101 min of MVPA at 4 METs per day, respectively. The average number of minutes per day spent in MVPA was similar for the longitudinal sample (137 and 96 min of MVPA per day in kindergarten and grade 2, respectively). Fifty-nine percent of the cross-sectional sample in kindergarten and 57% in grade 2 children met the Canadian 24-h movement guidelines. A slightly higher proportion of the longitudinal sample met the same guideline (63% in kindergarten and 60% in grade 2). Our daily MVPA levels were far higher than those found in studies of 3–5-year-old Scottish and Australian children, where time spent in MVPA ranged between 3% and 13% (approximately 23 min per day) [43,44,45]. Furthermore, MVPA levels in this study were almost twice those reported by Colley et al. [2] for Canadian 5-year-olds but similar in grade 2 to those in grade 4 reported by Nettlefold et al. [41]. The differences between our study and the national sample reflect where the data were collected. Similar to Nettlefold and colleagues [41], this study was conducted in British Columbia, Canada. In comparison to the majority of Canadian provinces, British Columbia has more sunshine and less snow during the year [43,46]. These environmental affordances may in part explain why the province has some of the lowest rates of obesity among the Canadian provinces and why MVPA levels are typically 10% higher than the national average [46,47]. Future research comparing year-round activity levels may provide valuable information about seasonal variation in physical activity and sedentary behaviour levels among children in Canada.

Notwithstanding the potential influence of weather on physical activity, our findings are similar to those of Janz and colleagues’ [21] tracking study that spanned 3 years (children 5–8 years of age). We found that MVPA declined from 22% to 16%, while the Janz et al. participants’ levels declined from 26% to 17%. These dramatic reductions in MVPA across the primary years were also evident in cross-sectional data from both Canada and the United States [3,25].

Sedentary behaviour minutes in our study were high. Specifically, children in kindergarten and grade 2 spent approximately 367 min and 439 min (60% and 72% of the day) engaged in sedentary behaviours, respectively. Similarly, the smaller longitudinal sample of children accrued approximately 366 min in kindergarten and 435 min in grade 2. In the present study, kindergarten children (cross-sectional and longitudinal) spent less time in sedentary behaviour in comparison to other Canadian 5-year-olds, whose daily average of sedentary behaviour was 381 min [2]. For older children, however, the 439 and 435 min of sedentary behaviour reported for the cross-sectional and longitudinal samples were similar [3]. Other studies examining Canadian children’s sedentary behaviour using accelerometers and identical cut-points differed from the findings of this study. Herman and colleagues [48] found much lower levels of sedentary behaviour (~360 min) among 8–10-year-old normal weight and overweight boys and girls. However, the authors indicated that these findings may not have been generalizable to the rest of Canada, without explaining their reasoning. Much higher levels of sedentary behaviour (539 min) have previously been found in somewhat older children in British Columbia [41]. It is possible that the higher rates of sedentary behaviour demonstrated by Nettlefold et al. are indicative of the age-related (8–11 years old versus 7 years old) increase in sedentary behaviour during childhood [2,3]. While there is no definitive reason why sedentary behaviour increases, perhaps school-time demands for sitting and recreational screen-time are greater in grade 2 than in kindergarten.

With each passing school year, there is an increase in workload, homework, and school-time sitting [23]. Efforts have been made to break up the amount of time in school children are sitting down. These efforts include standing desks [49] and physical activity breaks [50]. Outside of the school environment, watching television and other forms of screen-time have been directly linked to sedentary behaviour for children [4,48,51]. Strasburger [51] suggests that older children and adolescents spend more than 7 h per day engaged in various forms of screen-time and emphasized the amount of access youth have to sedentary pursuits. For example, in the United States it is estimated that 93% of youth aged 12–17 years have Internet access and over 70% have a cell phone. These studies illustrate the variety of factors contributing to sedentary behaviours among children and youth.

We used accelerometry to measure both physical activity and sedentary behaviour. While an effective measurement tool, there continues to be issues surrounding the classification of MVPA. Specifically, using cut-points that are too low may result in an overestimation of the amount of time spent in MVPA [28,29,37]. Research measuring physical activity intensity among children comparing both 3 MET and 4 MET values [31,37] revealed that using a more conservative approach to measuring MVPA (4 METs) yielded more accurate results for children [37]. We measured MVPA with both 3 METs and 4 METs to address this issue and enhance generalizability and the approach yielded differences in minutes accrued. In addition, as the Actigraph accelerometers that we used in this study do not assess the posture adopted when an individual is sedentary, we do not know what positions the children were adopting (e.g., standing, sitting, or lying) when they were sedentary, or whether they were using their upper body without moving their trunk. This potentially overestimates the level of sedentary behaviour; however, as the same protocol was adopted in kindergarten and in grade 2, the change in sedentary behaviour levels is unlikely to be an artifact but a real change in behaviour.

Our results showed an increase in physical activity levels by more than 30 min when using the 3 MET cut-point versus the 4 MET cut-point. These differences, when equated to minutes, can make a meaningful difference in classifying someone as achieving the health-related guidelines. Classification accuracy (e.g., meeting guidelines or not) has been shown to be influenced by the type of accelerometer used and how cut-points were developed [28]. Moving forward, it is important for consensus around classifying intensity among children, but until then, future research should classify moderate activity using both 3 MET and 4 MET cut-points.

We found that physical activity decreased while sedentary behaviour increased from kindergarten to grade 2. Among the longitudinal sample, this significant decrease equalled approximately 42 min of MVPA. Total physical activity decreased by more than 70 min while sedentary behaviour increased by almost 70 min in both the cross-sectional and longitudinal samples. These findings are consistent with previous research showing that physical activity significantly declines over time while sedentary behaviours increase [25,52]. It is clear from our kindergarten data that the capacity for higher levels of physical activity and lower levels of sedentary behaviour exists; however, we do not know why these dramatic changes occurred. Examining pressures during school and out-of-school time that may contribute to these changes is a next step in ameliorating these negative trends.

### 4.2. Limitations

This study is not without limitations. Perhaps the most significant was the rate of attrition in the longitudinal sample. Participants were excluded from the study if they did not have at least 10 hours of wear time for at least three weekdays and one weekend day. This resulted in a loss of 51% of the recruited participants from analysis. Further loss from the cross-sectional sample to the longitudinal cohort was due to factors such as: enrolment in a different school, consent obtained in kindergarten but not in grade 2 (or vice versa), or lack of wear time due to the belt not being comfortable. Despite this, the longitudinal sample results did not differ significantly from those derived from the larger cross-sectional sample and thus we believe that these findings are representative of our larger cross-sectional sample. This provides important information not only about the generalizability of the findings but also adds to the literature from a methodological standpoint. Although the sample was not sufficiently large to stratify these results by school (i.e., *n* = 8 schools), preliminary analyses revealed that school did not interact significantly with physical activity levels or sedentary behaviour.

## 5. Conclusions

Our findings suggest high levels of both MVPA and sedentary time among British Columbian children and that MVPA consistently declines while sedentary time increases from kindergarten to grade 2. It appears that there is a critical need to invest in strategies to maintain higher levels of physical activity across the primary years and reduce sedentary time, since these behaviours are associated with both positive and negative health outcomes, respectively. In addition, until consensus is reached, public health researchers should use both the 3 MET and 4 MET cut-points for MVPA in order to allow for more robust estimates and cross-study comparability.

## Figures and Tables

**Table 1:** Physical activity in minutes per day for the cross-sectional sample in kindergarten and grade 2.

Grade	Intensity (Minutes)	Mean	Std. Deviation	Minimum	Maximum
Kindergarten	Sedentary (<1.5 METs)	367.65	43.86	264.69	476.90
*n* = 75	MVPA 4 METs	133.17	29.00	65.55	210.50
	MVPA 3 METs	166.38	28.56	83.45	242.40
	Total physical activity	354.12	43.81	240.00	476.38
Grade 2	Sedentary (<1.5 METs)	443.21	58.68	322.06	642.30
	MVPA 4 METs	101.21	28.67	37.94	166.47
*n* = 73	MVPA 3 METs	135.20	28.32	69.76	192.30
	Total physical activity	291.78	42.65	193.39	463.67

MET: metabolic equivalent; MVPA: moderate-to-vigorous physical activity.

**Table 2:** Physical activity in minutes per hour for the cross-sectional sample in kindergarten and grade 2.

Grade	Intensity (Minutes)	Mean	Std. Deviation	Proportion per Hour	Minimum	Maximum
Kindergarten	Sedentary (<1.5 METs)	30.54	3.18	50.9	23.24	38.94
*n* = 75	MVPA 4 METs	11.08	2.44	18.5	5.35	17.82
	MVPA 3 METs	18.09	2.05	30.2	11.84	23.33
	Total physical activity	29.41	4.24	49.0	18.93	40.60
Grade 2	Sedentary (<1.5 METs)	36.26	3.59	60.4	29.38	44.31
*n* = 73	MVPA 4 METs	8.36	2.41	13.9	3.50	14.80
	MVPA 3 METs	14.22	2.99	23.7	7.82	19.20
	Total physical activity	23.73	4.57	39.6	12.76	35.23

**Table 3:** Longitudinal sample (*n* = 21) physical activity and sedentary behaviour per day.

Grade	Intensity (Minutes)	Mean	Std. Deviation	Minimum	Maximum
Kindergarten	Sedentary (<1.5 METs)	367.55	37.23	304.58	424.46
	MVPA 4 METs	136.99	28.27	80.45	193.05
	MVPA 3 METs	169.24	29.39	86.54	260.32
	Total physical activity	363.78	54.47	240.00	476.38
Grade 2	Sedentary (<1.5 METs)	435.33	47.16	348.55	505.69
	MVPA 4 METs	96.34	22.97	38.20	143.07
	MVPA 3 METs	132.21	26.22	63.67	190.03
	Total physical activity	291.88	42.70	210.00	367.42

**Table 4:** Longitudinal sample (*n* = 21) physical activity and sedentary behaviour per hour.

Grade	Intensity (Minutes)	Mean	Std. Deviation	Proportion per Hour	Minimum	Maximum
Kindergarten	Sedentary (<1.5 METs)	30.65	3.18	51.1	24.63	35.48
	MVPA 4 METs	11.39	2.26	19.0	7.10	15.79
	MVPA 3 METs	17.44	2.90	29.1	9.21	24.01
	Total physical activity	29.47	4.63	49.1	17.75	37.83
Grade 2	Sedentary (<1.5 METs)	36.31	3.16	60.5	30.78	42.26
	MVPA 4 METs	8.03	1.86	13.4	3.34	11.33
	MVPA 3 METs	14.18	2.21	23.6	6.19	20.54
	Total physical activity	23.68	3.97	39.5	15.70	30.76
